# Alterations of Sexual and Erectile Functions after Brachytherapy for Prostate Cancer Based on Patient-Reported Questionnaires

**DOI:** 10.1155/2024/5729185

**Published:** 2024-01-25

**Authors:** László Gesztesi, Zsuzsa S. Kocsis, Kliton Jorgo, Georgina Fröhlich, Csaba Polgár, Péter Ágoston

**Affiliations:** ^1^National Institute of Oncology, Centre of Radiotherapy, Budapest, Hungary; ^2^National Institute of Oncology, Centre of Radiotherapy, Department of Radiobiology and Diagnostic Onco-Cytogenetics and National Tumorbiology Laboratory, Budapest, Hungary; ^3^Semmelweis University, Department of Oncology, Budapest, Hungary

## Abstract

The aim of the study was to compare the side effects of high-dose-rate brachytherapy (HDRBT) and low-dose-rate brachytherapy (LDRBT), with a particular focus on the effects on sexual functions and sexual well-being (PROMOBRA study, NCT02258087). Localized low-risk and low-intermediate-risk prostate cancer patients were treated with mono LDR (*N* = 123, 145 Gy dose) or mono HDR brachytherapy (*N* = 117, 19/21 Gy). Prior to the treatment and during follow-up (at 3, 6, 9, 12, 18, and 24 months after treatment, and then annually after two years), patients completed patient-reported outcome measurement (PROM) questionnaires EORTC QLQ-PR-25, International Index of Erectile Function (IIEF), and IIEF-5 (SHIM). We compared the patients in different group breakdowns (HDR vs. LDR, hormone naïve and hormone-receiving HDR vs. LDR, hormone naïve and hormone-receiving patients in general, and 19 Gy HDR vs. 21 Gy HDR). In the hormone-naive LDR group, erectile function, orgasm function, sexual desire, satisfaction with intercourse, and overall satisfaction functions significantly decreased compared to baseline throughout the whole follow-up period. However, there were significant decreases in function at a maximum of three time points after HDR therapy without hormone therapy. In hormone-receiving patients, the orgasm function was significantly better in the HDR group at multiple time points compared to the baseline, and sexual desire improved at four time points. According to our results, both LDRBT and HDRBT can be safely administered to patients with localized prostate cancer. In hormone-naive patients, the HDR group showed only recovering decreases in sexual functions, while the LDR group showed a lasting decline in multiple areas. Thus, HDR appears to be more advantageous to hormone-naive patients.

## 1. Introduction

Prostate cancer (PC) is the third most common malignancy after skin cancer and lung cancer and the fifth most common cause of cancer-related mortality in men. In 2018, PC was responsible for 0.359 million deaths worldwide. While PC mostly affects the population above 55 years, men over 65 years are predisposed by 65% to the disease causing a great deal of concern [1, 2].

The treatment of prostate cancer patients depends on numerous factors including the stage of the tumour, life expectancy, and the prognostic group [3]. PC may be organ-confined, locally advanced, locoregionally advanced (with positive pelvic lymph nodes), or metastatic. For nonmetastatic PC, the most commonly used risk group system is the one introduced by D'Amico et al. [4].

In the management of nonmetastatic prostate cancer, brachytherapy (BT) is one of the possible curative treatment modalities. Both low-dose-rate (LDR) and high-dose-rate (HDR) treatment techniques are used as curative treatment, either as monotherapy or in combination with external beam irradiation [5]. LDR brachytherapy with permanently implanted I125 “seeds” is a widely used and well-proven method in the treatment of patients with low-risk or selected intermediate-risk, organ-confined prostate cancer [6–10]. The original use of prostate HDRBT was as a boost dose to raise the dose given to the prostate amending the external beam therapy [11–14]. Later, HDRBT was used as monotherapy in several centres and it has proven its value as a monotreatment modality for prostate cancer [15–22].

While both BT modalities are widely considered as safe and effective treatments, short- and long-term adverse effects can still occur after interventions. The most common types are genitourinary (GU) and gastrointestinal (GI) side effects, but a developing erectile dysfunction (ED) can also considerably impact the patients' quality of life and potentially can be the motive behind the patients choosing BT over surgery [23]. Combining with the effect of androgen deprivation therapy (ADT), which can be administered based on prevailing risk factors, typically elderly PC patients experience ED to some degree in a significant number of cases [24].

In our current paper, we wanted to focus on the impact of side effects, caused by either of the two types of prostate BT, on sexual function and sexual health and also how this influences the subjective well-being of the treated patients. The statistics were generated through prospective data collection from patients of a randomized trial initiated in 2015 and concluded in 2022, in which we compared one fraction of HDRBT and LDRBT (NCT02258087). The planned secondary endpoint of the study was the evaluation of quality of life (the primary endpoints are to be published separately). The short- and long-term side effects were compared for LDRBT and HDRBT monotherapy and were analysed in various subgroups.

The possibility of more than one treatment option (including active surveillance), makes our effort important to collect more information about the probability of various side effects to help future PC patients to make an informed decision about the treatment type that is going to fit their needs and priorities best.

## 2. Patients and Methods

Between January 2015 and December 2021, 250 patients with organ-confined, low-risk, or selected intermediate-risk prostate cancer were treated with BT, as monotherapy. 10 patients were excluded because they did not complete the sexual function follow-up questionnaires. 123 patients received LDR and 117 patients were treated with HDRBT administered in one fraction.

Patients with organ-confined, histologically proven adenocarcinoma of the prostate were selected for the study. The clinical stage had to be between cT1b and cT2c, Gleason score 3 + 3 or 3 + 4, and PSA level </ = 15 ng/ml. Patients had to be younger than 75 years and with a performance status Eastern Cooperative Oncology Group (ECOG) of 0 or 1. The rate of the positive biopsy tissue had to be less than 50% of the sample. All patients had previous imaging of the pelvic area (CT or MRI or both) and selected intermediate-risk patients underwent bone scans too.

All patients underwent a pretreatment transrectal ultrasound (TRUS) examination to prove that they were anatomically fit for BT. Patients with pubic arc interference, or with a prostate larger than 60 cm^3^, or with a distance between the rectum and prostate of less than 5 mm, were excluded. Exclusion criteria also included clinical stages of T3-T4, Gleason score of 4 + 3 or higher, PSA of >15 ng/mL, evidence of positive lymph node or distant metastases, previous radiation therapy to the pelvic region, or transurethral resection of the prostate in patient history. Patients who had severe dysuria and more than 15 points on the International Prostate Symptom Score (IPSS) were also excluded so as patients who were considered very low-risk: 3 or fewer positive biopsy samples where the malignant part was less than 50% of the whole sample, and PSA density was lower than 0.15 ng/ml.

Of the 240 patients, 81 (34%) had low-risk and 159 (66%) had selected intermediate-risk PC. 123 patients (51%) received LDRBT and 117 (49%) received HDRBT. The median age was 66 years. Concerning the mean age, TNM status, Gleason score, mean iPSA (initial PSA), and the ratio of patients receiving neoadjuvant ADT, the study arms were balanced (Table 1). The duration of ADT was 3–6 months. No patients received hormonal treatment after the implantation.

Subjects were randomized to one of the two treatment arms using stratification by risk groups. Baseline evaluation included taking patient history and performance status and a physical examination with digital rectal examination, as well as pretreatment PSA. Before the treatment and during the follow-up, the patients filled out the EORTC QLQ-PR-25, the International Index of Erectile Function (IIEF), and IIEF-5 (SHIM) questionnaires [25–27]. The questionnaires were completed by the patients before the treatment and at 3, 6, 9, 12, 18, and 24 months and then yearly after the treatment.

### 2.1. HDRBT Arm

The HDRBT was performed in spinal anaesthesia in the lithotomy position with a Foley catheter inserted into the urethra with transrectal ultrasound (TRUS) (Pro Focus 2202; BK Medical ApS, Herlev, Denmark) guidance. Two fixation needles were used to decrease the longitudinal movement of the prostate during needle insertion. Then, the needles were inserted while being monitored by live longitudinal TRUS image and an intraoperative plan was made by using the images taken after the needle insertion. The prescribed dose for the prostate was 19 Gy for 48 patients, and then we increased the dose to 21 Gy as the first group of patients experienced very mild side effects. The Oncentra Prostate 3.2.2 (Elekta Brachytherapy, Veenendaal, The Netherlands) treatment planning system was used for treatment planning based on TRUS images (5 mm intervals). After manual preplanning of the position of the metal needles, the HIPO (hybrid inverse planning and optimization) inverse optimization algorithm was used to determine the dwell times of the Ir-192 source. For dose calculation, the TG-43 formalism was used.

### 2.2. LDRBT Arm

BEBIG stranded seeds (BEBIG Medical GmbH, Berlin, Germany) were implanted. SPOT PRO 3.1 and Oncentra Prostate 3.2.2 (Elekta Brachytherapy, Veenendaal, The Netherlands) treatment planning systems were used. To optimize the seed positions, the inverse planning simulated annealing (IPSA) algorithm was used, and then the optimal solution was reached with manual modifications. Live TRUS guidance was used for the seed implantation. The prescribed dose was 145 Gy to the surface of the prostate. 4 weeks after the implantation, the postimplantation plan was created and evaluated on a CT or on a CT-MRI fusion.

### 2.3. Questionnaires

**Figure 1:**
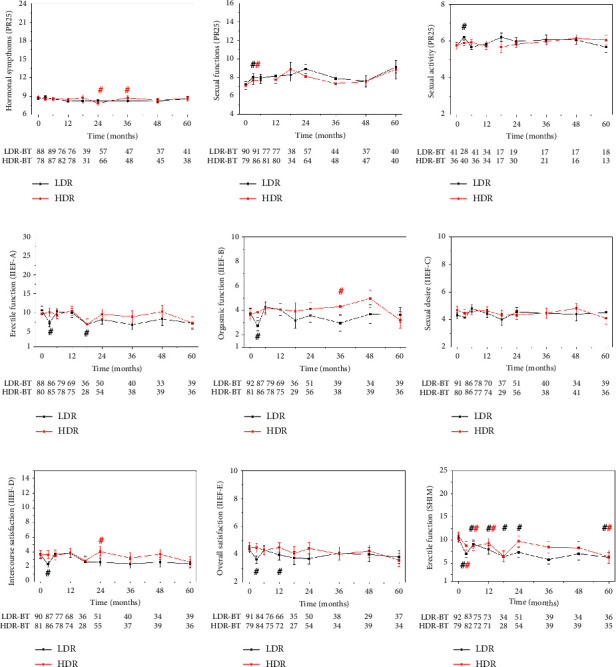
Sexual functions at follow-up points in the LDRBT (black) and HDRBT (red) groups. The *y*-axis of all graphs is scaled according to the points achievable of the given question group. Significant differences between LDRBT and HDRBT groups are indicated by asterisks, and significant differences from baseline are indicated by double crosses in the corresponding colour. Sample sizes of the comparison groups at every time point are shown under the plots.

**Figure 2:**
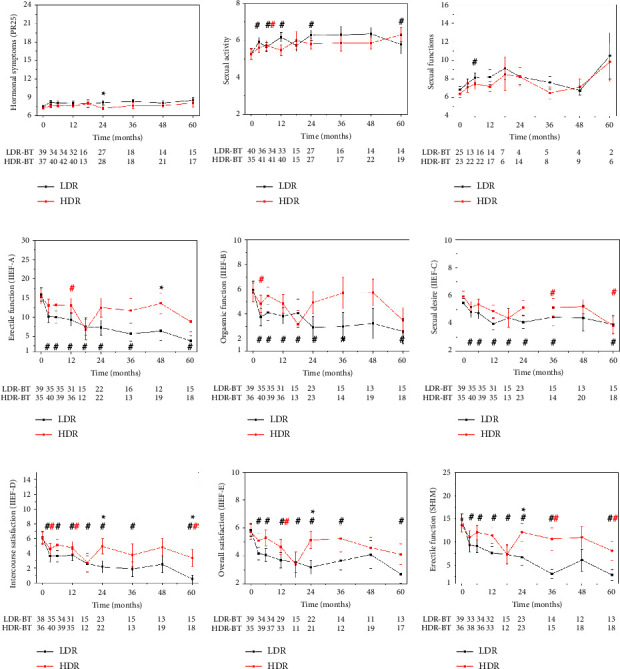
Sexual functions at follow-up points in the LDRBT (black) and HDRBT (red) groups of the hormone naïve patients. The *y*-axis of all graphs is scaled according to the points achievable of the given question group. Significant differences between LDRBT and HDRBT groups are indicated by asterisks, and significant differences from baseline are indicated by double crosses in the corresponding colour. Sample sizes of the comparison groups at every time point are shown under the plots.

**Figure 3:**
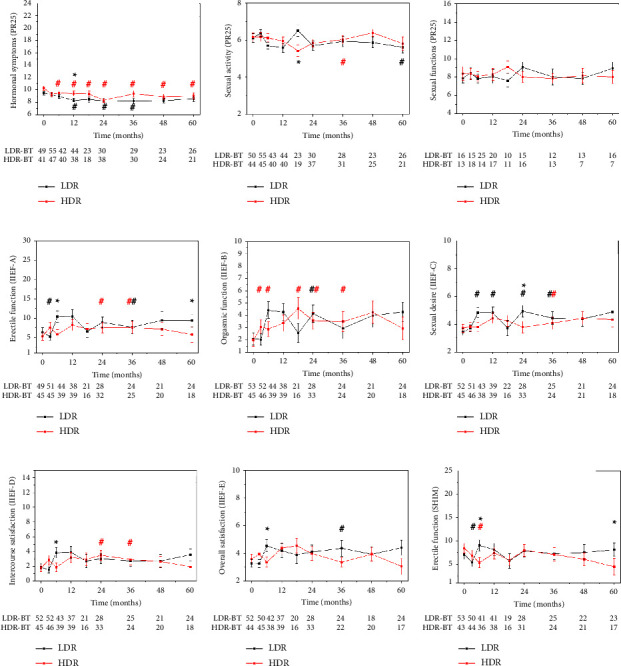
Hormonal symptoms, orgasmic function, and sexual desire at follow-up points in the LDRBT (black) and HDRBT (red) groups of the hormone-receiving patients. The *y*-axis of all graphs is scaled according to the points achievable of the given question group. Significant differences between LDRBT and HDRBT groups are indicated by asterisks, and significant differences from baseline are indicated by double crosses in the corresponding colour. Sample sizes of the comparison groups at every time point are shown under the plots.

**Figure 4:**
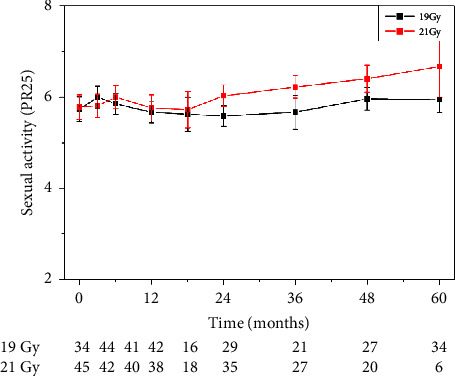
Sexual activity scores at follow-up points in the 19 Gy HDRBT (black) and 21 Gy HDRBT (red) groups. Sample sizes of the comparison groups at every time point are shown under the plot.

Groups of questions were formed to assess the various symptom groups in PR-25 and IIEF as described earlier (PR-25 [26] and IIEF [25]). The sum of scores was used in the case of the SHIM questionnaire [28]. The 20–22 questions of the PR-25 questionnaire were assessed in reverse as published before [29], as in these cases, higher scores indicated favourable outcomes, while in other questions, higher scores reflected more severe side effects (Table 2). We indicated the number of the completed questionnaires in the different analysis groups under every comparison plot (Figures 1–4).

### 2.4. Statistical Analysis

The Mann–Whitney *U* test was used for comparing the PR-25, SHIM, and IIEF test results of the treatment groups. The Wilcoxon matched pairs test was performed for comparison of the scores at given time points and baseline. The Cronbach alpha analysis was performed to test the reliability of the questionnaires, and we considered values above 0.7 to be acceptable. More than 10% of the achievable score deviation from baseline was interpreted as a clinically significant change in questionnaire score means. OriginPro 8.5, GraphPad Prism (San Diego, CA, USA), and STATISTICA 7 (StatSoft, Tulsa, USA) were used for calculations and presentation of the data. The *y*-axis of all graphs was scaled according to the points achievable of the given question group.

## 3. Results

240 patients were randomized in our study, that is, 123 patients in the LDRBT arm and 117 in the HDRBT arm. Some patients filled out the questionnaires only partially, and completeness was 77–84% (of the patients, who reached five-year follow-up) five years after the therapy. In the case of the sexual function in the question group (PR-25, questions 22–25), however, 32% of the patients answered before therapy and 33% five years later. The patients who answered varied, but for calculating mean values, all answers were used to compare BT techniques.

### 3.1. Effect of BT Modalities

When analysing the treatment effects on sexual activity (questions 20-21) and sexual functions (questions 22–25) in the question groups of the PR-25 questionnaire, no significant difference was found between the HDR and LDR arms (Mann–Whitney test) in any of the time points. Based on the IIEF questionnaires, erectile function (IIEF questions 1–5 and 15), orgasmic function (questions 9-10), sexual desire (questions 11-12), intercourse satisfaction (questions 6–8), and overall satisfaction with sexual life (questions 13-14) were evaluated. Neither of these, nor the SHIM questionnaire (Sexual Health Inventory for Men) showed a significant difference at any of the follow-up points between the HDR and LDR arms, although the HDR arm tended to have superiority everywhere (Figure 1).

It can be meaningful to evaluate the changes at follow-up points compared to the baseline level, to see if any of the treatment types has worsened the values (Figure 1.). We applied Wilcoxon analysis, which compared every patient's score with their own baseline score. The plots show the mean scores at each follow-up point, but the Wilcoxon analysis only uses scores with baseline pairs (double crosses indicate significant differences from baseline).

In most question groups at 3 month-time points, an acute spike can be seen with worse values for the LDR arm which balances out later. It can be considered a clinically significant decrease in IIEF (A, B, D, and E) and SHIM. In the SHIM questionnaire of the LDR arm, there was a significant decline in erectile function compared to baseline at not only after 3 months but also at 6, 12, 18, 24, and 60 month-time points (double crosses on Figure 1). Additionally, in the HDRBT arm, SHIM values at 3, 6, 12, and 60 months showed a decrease compared to values before therapy.

### 3.2. Subpopulation Based on Hormone Therapy (HT)

The results of PR-25 were evaluated in two subpopulations: hormone-naive and hormone-receiving.

### 3.3. HT-Naive

The hormone-naive group experienced a significant decrease only in sexual activity (in the PR-25 question group) in the case of HDRBT, at six months. However, in the LDR group, the sexual activity decreased at 3, 6, 12, and 24 months (Figures 2(a)–2(c)). In the LDRBT group, the IIEF symptom scores (erectile function, orgasmic function, sexual desire, intercourse satisfaction, and overall satisfaction) significantly decreased throughout the entire follow-up compared to the baseline (Figures 2(d)–2(i)).These decreases were also clinically significant. This indicates that without the suppressing effect of hormone therapy on sexual functions, HDRBT proved to be superior.

Statistically, there were only a few significantly different scores between LDR and HDR groups in the case of the hormone-naive patients. The IIEF-D, IIEF-E, and SHIM scores of the HDRBT-treated patients had a better sexual function (higher scores) than the scores of the LDRBT-treated patients at 24 months (Figures 2(g)–2(i)).

### 3.4. Hormone-Receiving Patients

Compared to baseline, the hormone-receiving group showed no significant improvement in sexual side effects (sexual activity and sexual functions) measured by the PR-25 questions (Figures 3(b) and 3(c)). In the group receiving androgen deprivation therapy (ADT), side effects caused by hormones (PR-25, questions 14–19: hot flushes, leg swelling, nipple sensitivity, weight gain/loss, and feeling of masculinity) resolved in both brachytherapy groups compared to baseline. This improvement began approximately at 1 year after treatment in the case of LDRBT and at 3 months in the HDRBT group (Figure 3(a)).

In hormone-receiving patients, the scores of orgasmic function improved in the HDR group at 3, 6, 18, 24, and 36 months (Figure 3(e)). Additionally, in the LDR group, the sexual desire was better at 6, 12, 24, and 36 months than at baseline (Figure 3(f)). In the SHIM scores, there was an acute worsening in both treatment arms. The differences between LDRBT and HDRBT arms seem to be clinically irrelevant as they only concern a few single follow-up points.

### 3.5. Difference between HT-Naive and HT-Receiving Patients

The baseline points for hormone-naive patients were significantly better than those for hormone-receiving patients as the latter group started hormone therapy months before BT, thus worsening the baseline score (Supplementary Figures 1 and 2).

In the LDRBT group, sexual activity, orgasmic function, and overall satisfaction were worse only in the ADT-receiving group at baseline (Supplementary Figures 1B, 2B, and 2B). Furthermore, between HT-naive and HT-receiving patients, sexual function was not significantly different after radiotherapy due to the received hormone (Supplementary Figure 1C). Erectile function, sexual desire, intercourse satisfaction, and SHIM erectile function differed for 3 months and hormonal symptoms persisted for 6 months (Supplementary Figures 2A, 2C, 2G, and 1A) (we interpret differences at 60 months as statistical mistakes of not enough data.)

In the case of HDRBT patients, PR-25 hormonal symptom scores differed for 48 months and sexual activity and function differed only at baseline (Supplementary Figures 1D–1F). IIEF and SHIM scores mostly decreased due to HT for 6 months (Supplementary Figures 2D–2F and 2J–2L).

### 3.6. Dose Dependence in HDRBT

No significant differences were found regarding side effects comparing 19 and 21 Gy in the HDR group. Furthermore, we could not reveal any significant difference between scores before and after 19 or 21 Gy HDR therapy (pairwise Wilcoxon analysis) (example in Figure 4).

### 3.7. Scores of the Different Question Groups and Questionnaires

We analysed the reliability of the questionnaires by questionnaire groups with Cronbach's alpha test (Table 3). The hormonal symptoms question group in PR25 questioonaire showed less than 0.7 Cronbach's alpha (0.425) and poor interitem correlation, which means the responses to the different questions in the question groups were not consistent. We tested which question had no significant correlation with the sum of the question group (data not shown). We observed that question 17 (has weight loss been a problem for you) was responsible (*p* = 0.081) for the inconsistency. The Cronbach's alpha of sexual function was also 0.632 with an interitem correlation of 0.301, but all questions correlated significantly well with the sum of the question group. The reliability of the IIEF question groups and SHIM was excellent in our analysis.

## 4. Discussion

The 5-year survival rate of men treated (radiotherapy, surgery, and HT) with organ-confined prostate cancer is high: around 95–97.6% [26, 30, 31]. Therefore, the quality of life after treatment, sexually and other, becomes a particularly important issue. Providing the opportunity of an informed choice between different treatment modalities is highlighted as each of the common treatment modalities such as radical prostatectomy, external beam RT (with gantry or robotic arm), and BT can cause adverse effects on various facets of sexual health [32]. This relevance is emphasized by the fact that many patients are willing to trade off survival from prostate cancer for a higher likelihood of satisfying potency after treatment [5].

Erectile dysfunction and sexual quality of life are of course influenced by several other factors as well, such as age; comorbidities, such as diabetes or hypertension; benign prostatic hyperplasia; decreased initial potency; habitual factors, such as smoking; and even the dose to the apex of the prostate and to the penile bulb [33–35]. It should be noted here that not all sources found a correlation between ED (after LDRBT) and penile bulb dose, pretreatment potency, age, or diabetes [36].

One of the important factors influencing ED among patients treated with prostate cancer is ADT and its role was investigated in our current study. 44% of our patients received ADT, as a cytoreductive or neoadjuvant hormone therapy before implantation. Following cytoreductive ADT, the prostate volume shrank enough to make the patient fit for BT [37]. For some other patients, ADT was started because of the recommendations for intermediate-risk disease [38]. Whichever the cause was, adding ADT increased the prevalence and frequency of sexual complaints before BT. Also, it was seen that the HT-receiving patients reached the IIEF and SHIM scores of patients without ADT approximately after three months in the LDR arm and mostly after 6 months in the HDR group (Supplementary Figures 1 and 2).

Whether or not to administer ADT, and for how long is highly controversial because besides its various positive effects, it also has several side effects. It may elevate the risk of fatal and nonfatal cardiovascular events especially when Luteinizing hormone-releasing hormone (LHRH) analogues are used. Surgical castration and antiandrogen monotherapy seemed to have a lower impact on cardiac function [39–41]. ADT can also lead to an increased risk of developing diabetes due to reducing the insulin receptor sensitivity, especially when administered for a longer time [40, 42, 43]. Another well-known side effect of ADT is the bone mineral density reduction [44], which may lead to osteoporosis and eventually to elevated risk of fractures [45, 46]. However, theincreased health risks were more prominent in patients receiving long-term HT than in patients recieving short-term HT, like our study participants. [47, 48].

There were hormonal treatment adverse effects (PR-25 14–19) observable for 6 months in the LDRBT group and 48 months in the HDRBT group (Supplementary Figures 1A and 1D). The reason for this phenomenon is unclear, and we could not find any explanation in the literature. Thus, the possibility of statistical coincidence emerges.

Sexual activity and sexual functions were reduced by ADT only at baseline in both brachytherapy arms (Supplementary Figure 1). The LDRBT arm showed worse adverse effects due to hormone therapy for less time in IIEF and SHIM scores. However, in the HDR group, the difference could be seen even until 18 months (sexual desire). Interestingly, the seed implantation delivers the dose over a period of one year, and approximately 65% of the dose was administered till the effects of the ADT resolved (Supplementary Figure 2).

In hormone-naive patients, a significant decrease in sexual function was observed with a comparison of actual and baseline scores (Figure 2). In the LDRBT group, all IIEF and SHIM scores differed throughout the whole follow-up, while in the HDRBT group, most scores differed only at one or two follow-up points compared to the baseline (Figure 2). This suggests that without giving hormone therapy, HDRBT seems to have milder side effects on sexual functions than LDRBT.

For hormone-receiving patients, time-dependent worsening of hormonal effect scores (PR-25 14–19 questions) was dominant (Figure 3). These effects were mild, but clinically significant.

The scores for sexual side effects after 21 Gy of HDRBT tended to be worse but did not differ significantly from scores after 19 Gy as demonstrated on the sexual activity in the question group in Figure 4.

The rates of answered questionnaires of sexual function were lower than the completeness of other questionnaires because only sexually active patients were asked to answer them. In the same study on the same patients, GI and GU questionnaires were answered with a higher rate (97% in the case of IPSS questionnaires), and it highlights the problem of patient's reluctance of discussing their sexual life/problems. Emphasized education and stricter control of patient follow-up with such types of questionnaires can be suggested. Shorter and less complicated questionnaires might have helped increase the answering rate and making such surveys available online could make the patients more willing to answer.

As was mentioned before, the 20–22 questions of the PR-25 questionnaire were assessed in reverse, as in these cases, higher scores indicated favourable outcomes, while for other questions, a higher score meant more severe side effects. This reverse scaling was mentioned in the literature [29], and it indicates a mistake in the PR-25 questionnaire that should be corrected, so researchers can easily interpret the statistics and they would not need this correction for the statistical analysis. The Cronbach alpha test showed that the hormonal treatment in the question group of the PR-25 questionnaire is less valid as shown before (49). In our opinion, question 17 (has weight loss been a problem for you) is hard for the patients to interpret. This question statistically was proven to be responsible for low interquestionnaire consistency.

An important issue is to compare side effects after BT with the side effects caused by External Beam Radiation Therapy (EBRT) techniques, such as intensity-modulated normofractionated or hypofractionated radiotherapy or radiotherapy with CyberKnife. The short treatment time of stereotactic radiotherapy makes it the most “popular” alternative of BT. With this information, patients would have comprehensive information on their choice of therapy and its possible efficiency and side effects. We aim to facilitate informed decisions with the help of Table 4, where we show our classified results of the erectile dysfunctions according to the elapsed time after BT.

In the retrospective study of Rana et al. [49], 102 nonmetastatic patients treated with SBRT using CyberKnife (5 times 7-8 Gy) at a single institution were evaluated. The SHIM score decreased significantly at 1 month after treatment from the baseline value of 13.52 to 11.95 (*p*  < 0.001) and continued to decrease below baseline at 1 year after treatment to 10.56 (*p*  <  0.001). The SHIM score started improving at 18 months but was still significantly less than the baseline at 12.12 (*p*=0.0100). After 2 years, the mean SHIM score did not significantly differ from the baseline at 12.57 (*p*=0.3400) and continued to improve after 3 years with a mean SHIM score of 13.06.

In the review article presented by Loi et al. [50], 12 studies were reviewed, but because of inconsistency in the definition of ED, they could not carry out a pooled analysis on this endpoint. A statistically significant decrease of scores in the sexual domains within 36 months of treatment was found in 5 of 12 studies, and in 5 of 12 studies, 26–55% of the patients developed ED at 60 months compared to baseline scores.

## 5. Conclusions

We found no significant difference between LDRBT and HDRBT, so we cannot conclude the explicit superiority of one to another regarding the effect on sexual functions. In the first 3 to 6 months, LDRBT does have stronger side effects, although after this time frame, the difference balances out. In the SHIM Questionnaires, we found that in both the HDR and LDR arms, the sexual function decreased by the therapy.

Without any form of HT, we observed HDRBT to be superior regarding sexual function side effects based on the IIEF and SHIM questionnaires. Side effects caused by HT fade away as hormone-receiving patients' values reach the levels of hormone-naive patients in 3–6 months regarding sexual interest and erectile function. Also, in the HDR group, the recovery tended to be faster. However, nonsexual HT caused side effects (PR-25 14–19) to last much longer, even for 48 months or more. 21 Gy HDR monotherapy seems to be just as safe as 19 Gy HDRBT regarding sexual side effects.

## Figures and Tables

**Table 1:** Patient characteristics.

Variables	Low risk			Medium risk		
	LDR (41 patients)	HDR (40 patients)	*P*	LDR (82 patients)	HDR (77 patients)	*P*
Mean age (range)	63.9 (53–74)	65.1 (54–74)	0.333	66.2 (50–75)	65.8 (53–76)	0.559

T1c	13 (31.7%)	17 (42.5%)	0.433	15 (18.3%)	9 (11.7%)	0.194
T2a	22 (53.7%)	19 (47.5%)		19 (23.1%)	23 (29.9%)	
T2b	6 (14.6%)	3 (7.5%)		15 (18.3%)	18 (23.4%)	
T2c	0	0		29 (35.4%)	27 (35.0%)	
No data	0	1 (2.5%)		4 (4.9%)	0	

GS ≤ 6	39 (95.1%)	40 (100%)	0.157	42 (51.2%)	42 (54.5%)	0.675
GS 7	0	0		40 (48.8%)	35 (45.5%)	
No data	2 (4.9%)	0		0	0	

Mean iPSA (range)	8.2 (4.5–13.6)	8.0 (3.1–11.7)	0.678	9.6 (0.5–15)	9.6 (1.3–18.6)	0.991

HT 0/1	21/20	20/20	0.913	33/49	31/43	0.835
19 Gy/21 Gy	—	22/18		—	26/51	

Baseline clinical data of the patients: there was no significant difference in baseline characteristics between the study arms in any of the risk groups according to the chi-squared test (LDR = low-dose-rate brachytherapy; HDR = high-dose-rate brachytherapy; GS = Gleason score; HT = hormone therapy).

**Table 2:** Questionnaires applied in the study.

Questionnaire	PR-25			IIEF					SHIM
Questions	14–19	20-21	22–25	1–5 and 15	9-10	11-12	6–8	13-14	1–5
Interpretation	Treatment/hormonal symptoms	Sexual activity and interest intensity	Sexual function	Erectile function	Orgasmic function	Sexual desire	Intercourse satisfaction	Overall satisfaction	Erectile function

Short name	Hormonal symptoms	Sexual activity	Sexual function	IIEF-A	IIEF-B	IIEF-C	IIEF-D	IIEF-E	SHIM

Score achievable	6–24	2–8	4–16	1–30	1–10	2–10	0–15	2–10	1–25
	Higher score means worse function			Higher score means better function					Higher score means better function

**Table 3:** Reliability analysis results of the different question groups.

Questionnaire	PR-25			IIEF					SHIM
Questions	14–19	20-21	22–25	1–5, 15	9-10	11-12	6–8	13-14	1–5
Interpretation	Treatment/hormonal symptoms	Sexual activity and interest intensity	Sexual function	Erectile function	Orgasmic function	Sexual desire	Intercourse satisfaction	Overall satisfaction	Erectile function

Short name	Hormonal symptoms	Sexual activity	Sexual function	IIEF-A	IIEF-B	IIEF-C	IIEF-D	IIEF-E	SHIM

Score achievable	6–24	2–8	4–16	1–30	1–10	2–10	0–15	2–10	1–25
	Higher score means worse function			Higher score means better function					Higher score means better function
Cronbach's alpha	0.425	0.841	0.632	0.968	0.985	0.908	0.939	0.917	0.958
Interitem correlation	0.087	0.727	0.301	0.856	0.970	0.834	0.864	0.849	0.836

**Table 4:** Severity of erectile dysfunction (SHIM) after radiotherapy.

	Baseline		3 months		6 months		12 months		18 months		24 months		36 months		48 months		60 months	
	HT 0 (%)	HT 1 (%)	HT 0 (%)	HT 1 (%)	HT 0 (%)	HT 1 (%)	HT 0 (%)	HT 1 (%)	HT 0 (%)	HT 1 (%)	HT 0 (%)	HT 1 (%)	HT 0 (%)	HT 1 (%)	HT 0 (%)	HT 1 (%)	HT 0 (%)	HT 1 (%)
Severe ED	26.7	53.6	47.1	72.5	42.4	60.8	48.3	60.5	58.3	70.6	56.5	54.4	65.5	56.3	51.7	67.5	75.9	66.7
Moderate ED	13.3	13.4	7.1	7.7	9.1	9.5	6.7	7.9	12.5	8.8	4.3	12.3	10.3	12.5	17.2	5.0	3.4	15.4
Mild-moderate ED	13.3	17.5	12.9	7.7	15.2	14.9	15.0	7.9	12.5	17.6	13.0	17.5	6.9	18.8	0.0	10.0	3.4	2.6
Mild ED	21.3	13.4	18.6	7.7	16.7	6.8	13.3	14.5	12.5	0.0	6.5	12.3	3.4	10.4	6.9	10.0	6.9	7.7
No signs of ED	25.3	2.1	14.3	4.4	16.7	8.1	16.7	9.2	4.2	2.9	19.6	3.5	13.8	2.1	24.1	7.5	10.3	7.7

## Data Availability

The data that support the findings of this study are available from the corresponding author upon reasonable request.

## Supplementary Materials

Supplementary Materials Supplementary Figure 1: PR-25 scores at follow-up points in the hormone-naive (black) and hormone-receiving (red) groups. The *y*-axis of all graphs is scaled according to the points achievable of the given question group. Significant differences between hormone-naive and hormone-receiving patient scores are indicated by asterisks, and significant differences from baseline are indicated by double crosses in the corresponding colour. Sample sizes of the comparison groups at every time point are shown under the plots. Supplementary Figure 2: IIEF and SHIM scores at follow-up points in the hormone-naive (black) and hormone-receiving (red) groups for patients receiving LDRBT (A–C and G–I) and HDRBT (D–F and J–L). The *y*-axis of all graphs is scaled according to the points achievable of the given question group. Significant differences between hormone-naive and hormone-receiving patient scores are indicated by asterisks, and significant differences from baseline are indicated by double crosses in the corresponding colour. Sample sizes of the comparison groups at every time point are shown under the plots.
