# Supplementary figures and images for: Alterations of Sexual and Erectile Functions after Brachytherapy for Prostate Cancer Based on Patient-Reported Questionnaires
Source: Prostate Cancer. 2024 Jan 25;2024:5729185. doi: 10.1155/2024/5729185 (PMC10834089; doi:10.1155/2024/5729185)

## LDR BT

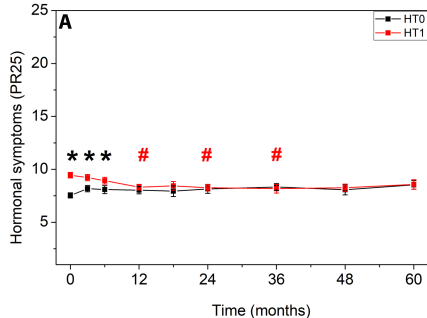

|      |    |    |    |    |    |    |    |    |    |
|------|----|----|----|----|----|----|----|----|----|
| HT 0 | 39 | 34 | 34 | 32 | 16 | 27 | 18 | 14 | 15 |
| HT 1 | 49 | 55 | 42 | 44 | 23 | 30 | 29 | 23 | 26 |

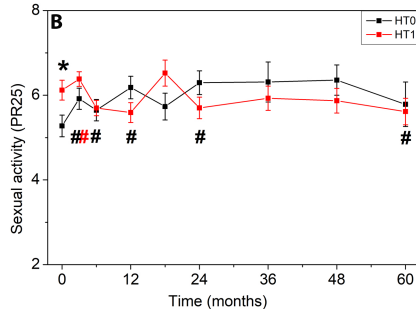

|      |    |    |    |    |    |    |    |    |    |
|------|----|----|----|----|----|----|----|----|----|
| HT 0 | 40 | 36 | 34 | 33 | 15 | 27 | 16 | 14 | 14 |
| HT 1 | 50 | 55 | 43 | 44 | 23 | 30 | 28 | 23 | 26 |

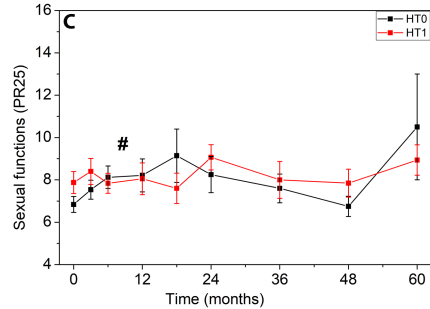

|      |    |    |    |    |    |    |    |    |    |
|------|----|----|----|----|----|----|----|----|----|
| HT 0 | 25 | 13 | 16 | 14 | 7  | 4  | 5  | 4  | 2  |
| HT 1 | 16 | 15 | 25 | 20 | 10 | 15 | 12 | 13 | 16 |

## HDR BT

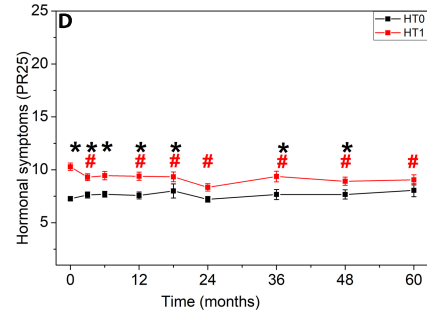

|      |    |    |    |    |    |    |    |    |    |
|------|----|----|----|----|----|----|----|----|----|
| HT 0 | 37 | 40 | 42 | 40 | 13 | 28 | 18 | 21 | 17 |
| HT 1 | 41 | 47 | 40 | 38 | 18 | 38 | 30 | 24 | 21 |

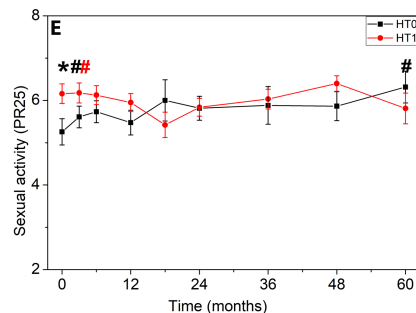

|      |    |    |    |    |    |    |    |    |    |
|------|----|----|----|----|----|----|----|----|----|
| HT 0 | 35 | 41 | 41 | 40 | 15 | 27 | 17 | 22 | 19 |
| HT 1 | 44 | 45 | 40 | 40 | 19 | 37 | 31 | 25 | 21 |

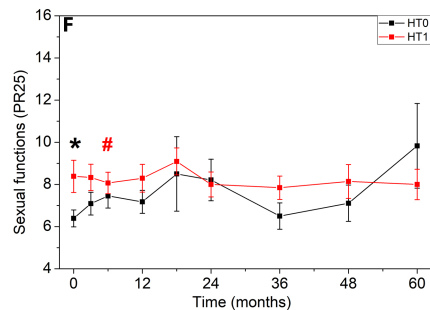

|      |    |    |    |    |    |    |    |   |   |
|------|----|----|----|----|----|----|----|---|---|
| HT 0 | 23 | 22 | 22 | 17 | 6  | 14 | 8  | 9 | 6 |
| HT 1 | 13 | 18 | 14 | 17 | 11 | 16 | 13 | 7 | 7 |

Supplement: Supplementary Materials — Supplementary Figure 1: PR-25 scores at follow-up points in the hormone-naive (black) and hormone-receiving (red) groups. The y-axis of all graphs is scaled according to the points achievable of the given question group. Significant differences between hormone-naive and hormone-receiving patient scores are indicated by asterisks, and significant differences from baseline are indicated by double crosses in the corresponding colour. Sample sizes of the comparison groups at every time point are shown under the plots. Supplementary Figure 2: IIEF and SHIM scores at follow-up points in the hormone-naive (black) and hormone-receiving (red) groups for patients receiving LDRBT (A–C and G–I) and HDRBT (D–F and J–L). The y-axis of all graphs is scaled according to the points achievable of the given question group. Significant differences between hormone-naive and hormone-receiving patient scores are indicated by asterisks, and significant differences from baseline are indicated by double crosses in the corresponding colour. Sample sizes of the comparison groups at every time point are shown under the plots. [file 5729185.f1.zip › Supplementary Figure1.pdf]
