# Supplementary material for: Alterations of Sexual and Erectile Functions after Brachytherapy for Prostate Cancer Based on Patient-Reported Questionnaires
Source: Prostate Cancer. 2024 Jan 25;2024:5729185. doi: 10.1155/2024/5729185 (PMC10834089; doi:10.1155/2024/5729185)
Supplement: Supplementary Materials — Supplementary Figure 1: PR-25 scores at follow-up points in the hormone-naive (black) and hormone-receiving (red) groups. The y-axis of all graphs is scaled according to the points achievable of the given question group. Significant differences between hormone-naive and hormone-receiving patient scores are indicated by asterisks, and significant differences from baseline are indicated by double crosses in the corresponding colour. Sample sizes of the comparison groups at every time point are shown under the plots. Supplementary Figure 2: IIEF and SHIM scores at follow-up points in the hormone-naive (black) and hormone-receiving (red) groups for patients receiving LDRBT (A–C and G–I) and HDRBT (D–F and J–L). The y-axis of all graphs is scaled according to the points achievable of the given question group. Significant differences between hormone-naive and hormone-receiving patient scores are indicated by asterisks, and significant differences from baseline are indicated by double crosses in the corresponding colour. Sample sizes of the comparison groups at every time point are shown under the plots. [file Supplementary Figure2.pdf › Supplementary Figure2.pdf]

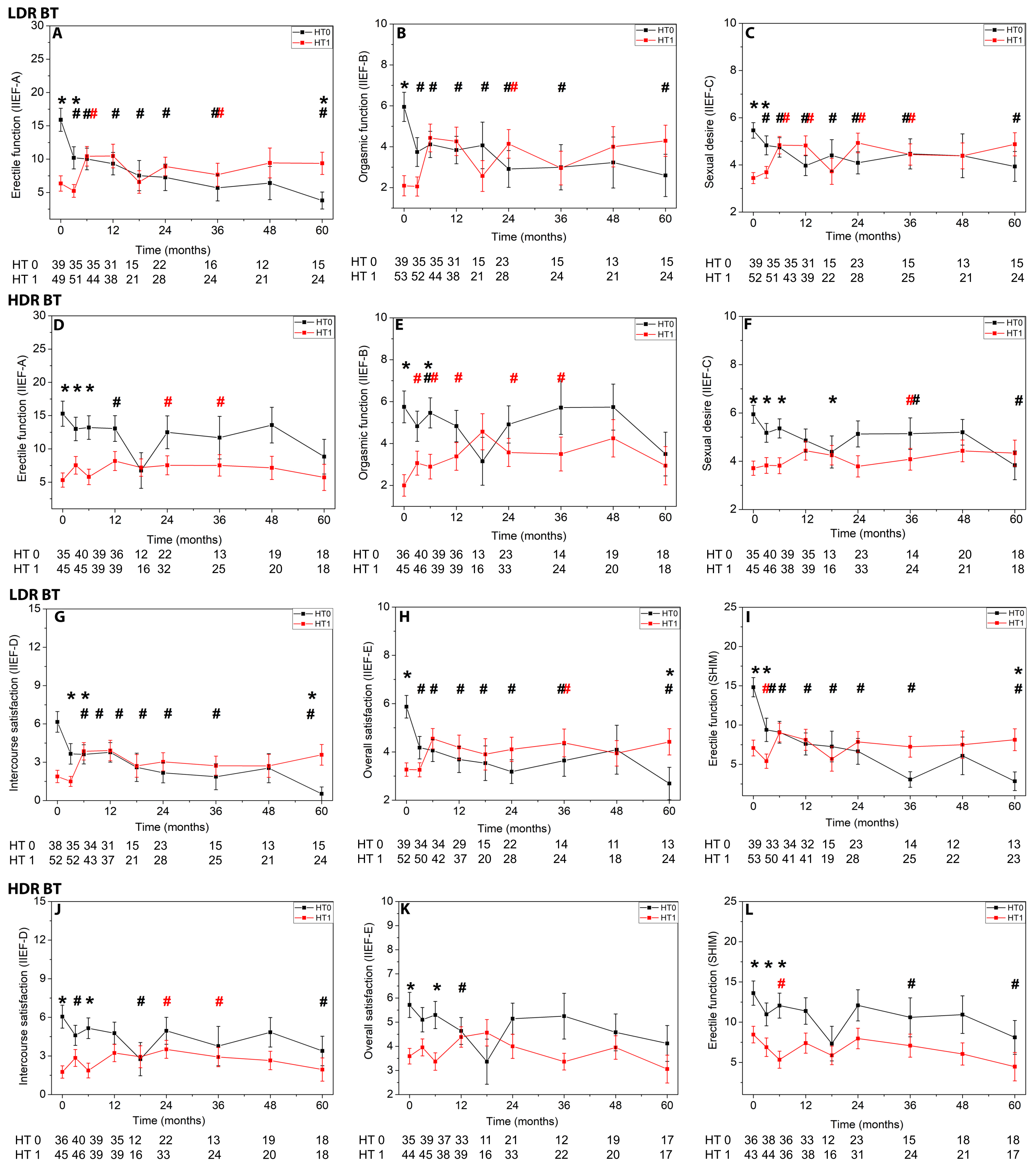

LDR BT
A
Erectile function (IIEF-A)
Time (months)
HT 0 39 35 35 31 15 22 16 12 15
HT 1 49 51 44 38 21 28 24 21 24
B
Orgasmic function (IIEF-B)
Time (months)
HT 0 39 35 35 31 15 23 15 13 15
HT 1 53 52 44 38 21 28 24 21 24
C
Sexual desire (IIEF-C)
Time (months)
HT 0 39 35 35 31 15 23 15 13 15
HT 1 52 51 43 39 22 28 25 21 24
HDR BT
D
Erectile function (IIEF-A)
Time (months)
HT 0 35 40 39 36 12 22 13 19 18
HT 1 45 45 39 39 16 32 25 20 18
E
Orgasmic function (IIEF-B)
Time (months)
HT 0 36 40 39 36 13 23 14 19 18
HT 1 45 46 39 39 16 33 24 20 18
F
Sexual desire (IIEF-C)
Time (months)
HT 0 35 40 39 35 13 23 14 20 18
HT 1 45 46 38 39 16 33 24 21 18
LDR BT
G
Intercourse satisfaction (IIEF-D)
Time (months)
HT 0 38 35 34 31 15 23 15 13 15
HT 1 52 52 43 37 21 28 25 21 24
H
Overall satisfaction (IIEF-E)
Time (months)
HT 0 39 34 34 29 15 22 14 11 13
HT 1 52 50 42 37 20 28 24 18 24
I
Erectile function (SHIM)
Time (months)
HT 0 39 33 34 32 15 23 14 12 13
HT 1 53 50 41 41 19 28 25 22 23
HDR BT
J
Intercourse satisfaction (IIEF-D)
Time (months)
HT 0 36 40 39 35 12 22 13 19 18
HT 1 45 46 39 39 16 33 24 20 18
K
Overall satisfaction (IIEF-E)
Time (months)
HT 0 35 39 37 33 11 21 12 19 17
HT 1 44 45 38 39 16 33 22 20 17
L
Erectile function (SHIM)
Time (months)
HT 0 36 38 36 33 12 23 15 18 18
HT 1 43 44 36 38 16 31 24 21 17
